# Propolis mitigates busulfan-induced testicular dysfunction in rats: insights into redox stabilization, PCNA modulation, and caspase-3 inhibition

**DOI:** 10.3389/fphar.2025.1656541

**Published:** 2025-10-13

**Authors:** Elham A. AbdAllah, Basant A. Eid, Hassan A. Hussein, Nasser S. Abou khalil, Sohair M. M. Ragab, Asmaa Y. Wahman, Hailah M. Almohaimeed, Rehab H. Moneeb, Hanem S. Abdel-Tawab, Zuhair M. Mohammedsaleh, Jameel Barnawi, Hanan S. A. Waly

**Affiliations:** ^1^ Zoology Department, Faculty of Science, New Valley University, New Valley, Egypt; ^2^ Department of Theriogenology, Faculty of Veterinary Medicine, Assiut University, Assiut, Egypt; ^3^ Department of Surgery, Obstetrics and Artificial Insemination, Faculty of Veterinary Medicine, Sphinx University, New Assiut, Egypt; ^4^ Department of Medical Physiology, Faculty of Medicine, Assiut University, Assiut, Egypt; ^5^ Department of Animal Biochemistry and Physiology, Faculty of Veterinary Medicine, Badr University, Assiut, Egypt; ^6^ Laboratory of Physiology, Department of Zoology and Entomology, Faculty of Sciences, Assiut University, Assiut, Egypt; ^7^ Chemistry Department, Faculty of Science, New Valley University, New Valley, Egypt; ^8^ Department of Basic Science, College of Medicine, Princess Nourah Bint Abdulrahman University, Riyadh, Saudi Arabia; ^9^ Laboratory of Molecular Cell Biology and Laboratory of Histology, Zoology and Entomology Department, Faculty of Science, Assiut University, Assiut, Egypt; ^10^ Department of Medical Laboratory Technology, Faculty of Applied Medical Sciences, University of Tabuk, Tabuk, Saudi Arabia; ^11^ Department of Medical Lab Technology, Prince Fahd Bin Sultan Research Chair, Faculty of Applied Medical Sciences, University of Tabuk, Tabuk, Saudi Arabia

**Keywords:** propolis, busulfan, testis, histopathology, antioxidants, apoptosis

## Abstract

**Introduction:**

Testicular cytofunctional defects are among the most hazardous effects of cancer chemotherapies. Propolis mitigates the fertility problems associated with gonadotoxic agents through its redox stabilizing, anti-apoptotic, and cytoprotective properties due to presence of bioactive agents identified in our study by gas chromatography–mass spectrometry analysis including, flavonoids, terpenes, aliphatic and aromatic compounds, and amino acids. Herein, we investigated the potential reversal effects of aqueous propolis on busulfan-induced reproductive abnormalities in adult rats.

**Methods:**

Thirty rats were randomly assigned to five experimental groups, with six animals per group, for duration of 6 weeks. The control group received only the vehicle daily through oral gavage. The DMSO group was given a single intraperitoneal injection of DMSO. The busulfan group received a single intraperitoneal injection of busulfan at a dose of 20 mg/kg body weight, followed by daily oral gavage. The propolis group was administered propolis daily via oral gavage at a dose of 100 mg/kg body weight. In the busulfan + propolis group, rats received a single intraperitoneal injection of busulfan at 20 mg/kg body weight, combined with daily oral gavage of propolis at a dose of 100 mg/kg body weight.

**Results and discussion:**

Busulfan exposure led to a decrease in serum levels of follicle-stimulating hormone, testosterone, and estradiol 17β, along with an increase in luteinizing hormone. It negatively affected sperm outcomes, causing a decline in sperm count and the percentages of live, normal, and motile sperm, while increasing the percentages of dead and abnormal sperm. Furthermore, busulfan disrupted the testicular defense system, as indicated by elevated testicular malondialdehyde levels and reductions in testicular nitric oxide and reduced glutathione levels, catalase and superoxide dismutase activities, as well as serum total antioxidant capacity. Marked histopathological changes were observed, in concomitant with strong immunoreactivity for proliferating cell nuclear antigen and caspase-3 in germ cells. Propolis supplementation effectively mitigated all these abnormalities in busulfan-intoxicated rats. Propolis is suggested as a potential complementary adjuvant for managing busulfan-induced reproductive dysfunction, owing to its reproductive hormone-modulating, redox-stabilizing, sperm-protective, and anti-apoptotic properties.

## 1 Introduction

Advancements in early detection and enhanced cancer treatment protocols have led to a significant rise in the number of young male cancer survivors (Miller et al., 2022). Fertility has emerged as a crucial aspect of quality of life for adult cancer patients (Drizin et al., 2021). Accordingly, preventing iatrogenic infertility resulting from chemotherapy has become a critical priority (Blumenfeld, 2012). Busulfan, one of the most spermicidal agents commonly utilized in treating lymphoma and chronic leukemia, is known to cause various side effects, including impairments to the reproductive system (Ezim and Abarikwu, 2023; Li et al., 2023). Experimental animal studies have shown that busulfan induces disturbances in testicular redox homeostasis, marked histological deterioration, and impaired semen parameters, along with disruptions in the sexual hormone profile (Abarikwu et al., 2022; Rostami et al., 2022; Ezim and Abarikwu, 2023; Li et al., 2023). Furthermore, busulfan administration resulted in a marked reduction in the proliferation/apoptosis ratio, characterized by decreased gene expression of proliferating cell nuclear antigen (PCNA) and increased gene expression of caspase-3 (Abd El-Hay et al., 2023).

Currently, semen cryopreservation prior to initiating cancer therapy remains the only reliable approach to safeguarding male fertility for the future (Liu et al., 2021). However, a considerable number of male cancer patients refrain from sperm cryopreservation for various reasons, including financial constraints, particularly given the need to fund potentially costly life-saving cancer treatments (Ledesma et al., 2023). Among these challenges, the importance of inexpensive natural materials emerges as an available option to address reproductive problems associated with cancer chemotherapy. Propolis, commonly referred to as bee glue, is a dark-brown, resin-like substance created by honeybees. It is typically formed by blending beeswax and bee saliva with exudates gathered from tree buds, sap flows, or other botanical sources (Hwang et al., 2020; Sforcin, 2016). It is rich in redox stabilizers, including flavonoids, cinnamic acid, gallic acid, stilbenes, catechins, and hydroxybenzoic acids, supporting its use in managing oxidative stress-related gonadal dysfunction (Kurek-Górecka et al., 2013; Moskwa et al., 2020; Wezgowiec et al., 2020; Laaroussi et al., 2021). A wide variety of literature confirmed the ability of propolis in counteracting the reproductive abnormalities associated with gonadal disruptors. Propolis mitigated the pathophysiological effects of cadmium on the testis by reducing histological damage to Leydig cells, Sertoli cells, and germ cells, enhancing the antioxidant capacity of the testis, restoring the balance of luteinizing hormone (LH), follicle-stimulating hormone (FSH), and testosterone output (Amr et al., 2024). In nicotinamide-streptozotocin-induced diabetic rats, propolis supplementation alleviated testicular damage by reducing the immuno-expression of proliferating cell nuclear antigen and enhancing DNA integrity in spermatogenic cells (Ashour, 2024). It also reduced caspase-3 activity and enhanced the proliferative rate in spermatogonial cells exposed to tert-butyl hydroperoxide and co-incubated with propolis (Duarte, 2024).

Thus, we hypothesize that propolis may enhance testicular cytofunctional characteristics in gonadotoxic chemotherapy models. Therefore, this study aimed to assess the impact of propolis on pituitary-gonadal hormone regulation, testicular antioxidant capacity, semen quality, and the immunoexpression of PCNA and caspase-3 in rats with busulfan-induced infertility.

## 2 Materials and methods

### 2.1 Busulfan preparation and propolis extraction

To prepare the busulfan solution, busulfan (Sigma-Aldrich Company, Catalog No. B2635, United States) was initially dissolved in dimethyl sulfoxide (DMSO). An equal volume of sterile water was then added, resulting in a final busulfan concentration of 20 mg/mL (Khosoroshahi et al., 2013). Propolis was procured from a local market. A total of 25 g of propolis powder was mixed with 250 mL of water at 40°C and kept on a shaker for 7 days in a dark room. Once fully dissolved, the suspension was filtered using clean filter paper, and the residue was re-extracted. The filtration process was repeated daily for 7 days. The final extract was dried using a rotary evaporator at 30°C–40°C (Matienzo and Lamorena, 2004).

### 2.2 Gas chromatography–mass spectrometry analysis

The chemical composition of propolis sample was analyzed using GC-MS. A 7890 N series gas chromatograph (Agilent Technologies), coupled with an Agilent 5,975 mass spectrometer selective detector (MSD) and fitted with an HP-5ms capillary column (30 m × 0.25 mm, 0.25 μm film thickness, Agilent Technologies), was employed for the analysis. The analysis was conducted at the Analytical Chemistry Unit (ACAL), Chemistry Department, Faculty of Science, Assiut University, Egypt. The extract was injected into the Agilent 7890-5975C GC/MSD system and desorbed at 250°C for 2 min. The injection port operated in splitless mode, with helium (99.999% purity) serving as both the vial pressurization and carrier gas at a flow rate of 1 mL/min. The oven temperature program began at 40°C, held for 2 min, and then increased at a rate of 10°C/min to 150°C, held for 6 min. It was further ramped at 10°C/min to 220°C, held for 6 min, and finally increased at 15°C/min to 280°C, where it was held for 15 min. The mass spectrometer operated in electronic impact mode at 70 eV, with the source temperature set at 230°C. Mass spectra were scanned in the range of m/z 30–600 amu at 1-s intervals. The chemical components of the propolis samples were identified using the National Institute of Standards and Technology (NISTW8N08) database. The mass spectral patterns were matched with the Wiley mass spectral library software (version 7n.1), installed on the computer linked to the GC/MS system, to determine the names, molecular weights, and structures of the detected compounds.

### 2.3 Experimental design

A total of 30 adult male Wistar albino rats, weighing 200 ± 10 g, were used in this experiment. The animals were procured from the Laboratory Animal House, Faculty of Medicine, Assiut University. The rats were acclimatized for 1 week prior to the experiment, during which they were provided with water and a standard commercial pellet diet. The animals were housed under controlled conditions, maintaining a temperature of 26°C ± 2°C, relative humidity of 40%–60%, and a 12-h light/dark cycle. After the acclimatization period, the rats were randomly divided into five experimental groups, each consisting of six animals. Control group received only the vehicle daily via oral gavage. DMSO group administered a single dose of DMSO intraperitoneally at dose of 0.2 mL (a 1:1 mixture of dimethyl sulfoxide and distilled water). The busulfan group received a single intraperitoneal injection of busulfan at a dose of 20 mg/kg body weight (Kean et al., 2002). Propolis group propolis was given orally via oral gavage at a dose of 100 mg/kg body weight daily (Baykalir et al., 2018). Busulfan + Propolis group received a single intraperitoneal injection of busulfan at a dose of 20 mg/kg body weight daily via oral gavage along with propolis at a dose of 100 mg/kg body weight (Kaya et al., 2019). After 6 weeks of the experiment, the rats in all groups were sacrificed under sodium thiopental anesthesia while fasting.

### 2.4 Sample collection

At the end of the 42-day experimental period, all animals were weighed and sacrificed under sodium thiopental anesthesia in a fasting state. Blood samples were immediately collected from each rat’s orbital sinus and placed in EDTA tubes. The blood samples were centrifuged at 4000 rpm for 10 min to separate the serum, which was then frozen at −80°C for subsequent biochemical analyses. The testes were carefully dissected and promptly rinsed with normal saline. The right testis was processed to obtain tissue homogenate, with the resulting supernatant stored at −80°C, while the left testis was fixed in a 10% formalin solution for histological analysis.

### 2.5 Measurement of pituitary-gonadal reproductive hormones and redox parameters

Luteinizing hormone (LH), follicle-stimulating hormone (FSH), testosterone, and estradiol 17β (E2) levels were determined using ELISA kits procured from Calbiotech Inc., United States (catalog numbers: LH231F, FS232F, TE373S, and ES380S, respectively). Malondialdehyde (MDA), nitric oxide (NO), catalase (CAT), superoxide dismutase (SOD), reduced glutathione (GSH), total antioxidant capacity (TAC), and total protein were measured using colorimetric kits (catalog numbers: MD 25 29, NO 25 33, CA 25 17, SD 25 21, GR 25 11, TA2512, TP 20 20) and provided by Biodiagnostic Company (Egypt). All redox parameters were normalized to total protein levels in the testicular homogenate. Hormonal measurements were performed using an ELISA reader (ELx800UV, BioTek Instruments, Inc., United States), while other biochemical analyses were conducted using a spectrophotometer (S1200, Unico, United States).

### 2.6 Semen analysis

To determine sperm count, the entire epididymis was minced in PBS medium and incubated at 37°C for 5 min. The sperm concentration was evaluated manually using a hemocytometer (Narayana et al., 2002). For evaluation of the viability of spermatozoa, fixed-smears were stained with Eosin (Carl Roth Gmbh + Co. KG, Karlsruhe, Germany)–Nigrosine (Sigma-Aldrich, Saint Louis, MO, United States) stain and examined at a ×400 magnification. A total of 300 spermatozoa were examined where colored/stained head spermatozoa were calculated as dead, and unstained ones as viable (Hussein et al., 2022). The percentage of morphologically abnormal spermatozoa was determined according to the method of Menkveld (2010). Motility was assessed using a percentage-based rating scale, as described by Kempinas et al. (1998). The integrity of sperm DNA was assessed using acridine orange (Varghese et al., 2011).

### 2.7 Histological inspection

Testis tissues were cut into slices approximately 3–4 mm thick and fixed in 10% neutral buffered formalin. The samples were then dehydrated through a series of graded ethanol concentrations, cleared in xylene, and embedded in paraffin. Paraffin blocks were sectioned using a microtome at a thickness of 4–6 μm and stained with Hematoxylin and Eosin (H&E) to examine the general tissue structure. The H&E-stained sections were observed under a Leica microscope (CH9435 Heerbrugg, Leica Microsystems, Switzerland).

### 2.8 PCNA and caspase-3 immunohistochemical analysis

Immunohistochemical analysis for PCNA and caspase-3 was performed following previously published protocols. Briefly, fixed rat testis samples were dehydrated in an ascending ethanol series and cleared in xylene. The tissues were embedded in paraffin, sectioned at a thickness of 5 μm, deparaffinized, and rehydrated. To block endogenous peroxidase activity, sections were incubated in 1% hydrogen peroxide in methanol for 30 min, rinsed, and incubated overnight in phosphate-buffered saline (PBS) containing a rat monoclonal antibody against PCNA (Dako, Milan, Italy) at a concentration of 1:250 in 10% bovine serum albumin (BSA). For caspase-3 analysis, sections were incubated with a rabbit polyclonal antibody (Cell Signaling Technology, Inc., Beverly, MA). Secondary antibodies, goat anti-mouse IgG (1:500) and goat anti-rabbit IgG, respectively, were applied in 10% BSA. The conventional avidin-biotin complex method was used to detect the reaction. Peroxidase activity was developed using a filtered solution of 5 mg of 3,3′-diaminobenzidine tetrahydrochloride (DAB; Sigma) dissolved in 15 mL of 0.05 M Tris buffer (pH 7.6) with 0.03% H_2_O_2_. Finally, the sections were mounted in a synthetic medium and examined under an Olympus light microscope, with imaging performed using accompanying software.

### 2.9 Statistical analysis

The data were presented as mean ± standard error of the mean (SEM). Statistical differences among groups were determined using one-way analysis of variance (ANOVA) followed by Duncan’s post hoc test. All analyses were performed using SPSS software for Windows, version 16.0 (SPSS, Inc., Chicago, IL, United States). A p-value of <0.05 was considered statistically significant.

## 3 Results

### 3.1 Bioactive components of propolis using GCMS

The GC–MS analysis of propolis identified 40 bioactive phytochemical compounds (Table 1). The predominant compounds detected were 2-hexadecanoyl glycerol (22.98%), beta-monosterine (9.87%), and tectochrysin (8.16%).

**TABLE 1:** Phytochemical compounds in propolis according to GC–MS analysis.

No	Chemical compound	Retention time (min)	Percent	Molecular formula	Molecular weight (g/mol)
1	Methyl 4-hydroxy-2-methoxy-3,5,6-trimethylbenzoate	8.81	0.763	C_15_H_18_O_5_	278.30
2	2,2,4,4,6,6,8,8-Octamethyl-1,3,5,7,2,4,6,8-tetraoxatetrasilocane	10.091	0.311	C_18_H_38_O_8_Si_4_	478.76
3	Butyl alcohol-2-d1	10.194	1.215	C_4_H_9_OH	74.12
4	1,3-Benzodiazol	10.447	0.733	C_7_H_4_N_2_	132.12
5	L-Cystine	10.634	0.314	C_6_H_12_N_2_O_4_S_2_	240.31
6	2-[(trimethylsilyl)oxy]-4-methylacetophenone	10.938	0.343	C_11_H_14_O_2_Si	218.36
7	4-Nitrochalcone	10.997	0.479	C_14_H_11_N_1_O_3_	239.25
8	2-Hydroxy-2-(4-hydroxy-3-methoxyphenyl)-acetic acid ethyl ester	11.385	0.169	C_14_H_18_O_5_	270.29
9	4-hydroxy-3-(2-oxo-2h-1-oxa-3-phenanthryl)-2(1h)-quinolinone	11.812	0.16	C_18_H_11_N_3_O_3_	309.30
10	Nonanoic acid, methyl ester	12.057	0.734	C_10_H_20_O_2_	172.27
11	2′,6′-Dihydroxyacetophenone, bis(trimethylsilyl) ether	12.995	0.188	C_14_H_18_O_4_Si_2_	294.44
12	7-Methoxy-2,3-diphenyl-4H-chromen-4-one	13.092	0.273	C_18_H_18_O_3_	278.34
13	Acetic acid, 2-phenylethyl ester	13.61	0.944	C_14_H_18_O_2_	222.29
14	N-(2-Hydroxybenzyl)alanine	14.464	0.621	C_9_H_13_NO_3_	81.21
15	Acetic acid, hydroxy-, ethyl ester	17.413	1.003	C_6_H_12_O_3_	130.16
16	α -curcumene	17.879	3.74	C_18_H_24_	244.37
17	1-Amino-3-(3,5-diiodo-2-methoxyphenyl) pyrido[1,2-a]benzimidazole-2,4-dicarbonitrile	18.727	0.947	C_13_H_8_N_4_I_2_O	404.02
18	D3-glycine	19.671	0.716	C_2_H_5_N_3_O_3_	103.13
19	(2-Oxido-1,3,2-dioxathiolan-4-yl) methanol	20.441	0.336	C_5_H_10_O_4_S	166.20
20	Guaiol	21.223	0.773	C_15_H_24_O	224.35
21	3-O-Methyl-D-glucose	22.116	12.545	C_7_H_14_O_7_	178.19
22	Asparagine	22.343	0.448	C_4_H_8_N_2_O_3_	132.12
23	β -Eudesmol	22.453	2.682	C_15_H_24_O	224.35
24	Griseoviridin	22.698	1.192	C_16_H_18_N_2_O_4_	302.33
25	3-ethyl-5- (2-ethylbutyl)- octadecane	23.293	0.408	C_18_H_38_	254.49
26	Guanosine	23.546	0.216	C_11_H_15_N_5_O_5_	283.24
27	Palmitic acid, methyl ester	26.405	4.693	C_16_H_32_O_2_	256.42
28	Atomoxetine	29.083	0.737	C_18_H_24_N_2_O	274.40
29	Octadecanoic acid, methyl ester	29.542	1.325	C_18_H_36_O_2_	284.48
30	Pinostrobin chalcone	34.834	7.793	C_15_H_14_O_3_	242.27
31	5-Methylindol	35.564	0.986	C_9_H_9_N	133.17
32	Pinocembrin	35.72	3.843	C_15_H_14_O_5_	270.27
33	2-Hexadecanoyl glycerol	35.862	22.976	C_18_H_36_O_3_	294.48
34	Methyl-n-octadecylamine	36.257	0.662	C_19_H_41_N	281.53
35	Tectochrysin	36.806	8.162	C_15_H_12_O_3_	244.25
36	Apigenin 7-methyl ether	37.22	1.409	C_15_H_12_O_5_	286.25
37	L-Alanine, N-propoxycarbonyl-, hexyl ester	37.492	0.809	C_14_H_19_NO_3_	251.31
38	Chrysin	37.731	2.014	C_15_H_14_O_4_	252.27
39	Beta-monostearin	37.88	9.865	C_18_H_36_O_4_	342.5
40	Glycyl-D-asparagine	38.152	1.024	C_6_H_11_N_3_O_4_	173.17

### 3.2 Propolis restored the reproductive hormones profile in busulfan-exposed rats

No significant differences were observed in serum levels of LH, FSH, testosterone, and E2 between the control group and the DMSO group. However, busulfan exposure in rats caused a significant reduction in FSH, testosterone, and E2 levels, accompanied by a marked increase in LH compared to the control group. A similar trend was observed when comparing the busulfan group with the DMSO group, except for FSH levels, which showed no significant difference between these groups. In the propolis group, a significant decrease in LH, testosterone, and E2 levels was noted compared to both the control and DMSO groups. FSH levels were significantly lower in the propolis group compared to the control group but showed no significant change when compared to the DMSO or busulfan groups. No significant differences were found in any of the measured reproductive hormones between the propolis group and the busulfan group, except for LH levels, which were significantly lower in the propolis group. Co-administration of propolis with busulfan significantly increased FSH, LH, testosterone, and E2 levels compared to the busulfan group, although these levels remained significantly lower than those of the control group, except for testosterone, which was restored to control levels (Table 2).

**TABLE 2:** Effects of propolis on the plasma levels of reproductive hormones in busulfan intoxicated rats.

Group Parameter	Control	DMSO	Busulfan	Propolis	Busulfan + propolis	P value
LH level (mU/mL)	1.580 ± 0.163^c^	1.790 ± 0.018^c^	2.379 ± 0.181^b^	0.930 ± 0.044^d^	2.924 ± 0.150^a^	0.000
FSH level (ng/mL)	3.125 ± 0.132^b^	2.650 ± 0.050^bc^	2.250 ± 0.112^c^	2.524 ± 0.185^c^	4.800 ± 0.389^a^	0.000
Testosterone level (ng/mL)	3.450 ± 0.225^a^	3.024 ± 0.295^a^	1.960 ± 0.243^b^	2.075 ± 0.248^b^	3.212 ± 0.198^a^	0.000
E2 level (Pg/mL)	12.220 ± 1.196^b^	11.900 ± 0.781^b^	3.200 ± 0.569^c^	3.360 ± 0.912^c^	20.237 ± 1.641^a^	0.000

FSH, follicle stimulating hormone; LH, luteinizing hormone; E2, estradiol-17β.

Data are expressed as the mean ± SE (n = 8).

^abc^ different letters indicate significant difference at P<0.05.

### 3.3 Propolis mitigated the negative effects of busulfan on sperm quantity and quality in rats

All measured semen parameters showed no significant differences between the control and DMSO groups, except for the percentage of live and normal sperm, which was significantly lower in the DMSO group compared to the control. Busulfan exposure had detrimental effects on semen quality, as evidenced by a significant reduction in sperm count, and the percentages of live, normal, and motile sperm, alongside a marked increase in dead and abnormal sperm percentages compared to both the control and DMSO groups. Propolis supplementation resulted in a significant decrease in the percentages of dead, live, normal, abnormal, and motile sperm, with no significant change in sperm count compared to the control and DMSO groups. However, in comparison to the busulfan group, propolis supplementation significantly improved semen quality, demonstrated by increased sperm count, and the percentages of live, normal, and motile sperm, together with a reduction in the percentages of dead and abnormal sperm. In busulfan-intoxicated rats, propolis administration significantly enhanced semen characteristics, as evidenced by increased sperm count, and the percentages of live, normal, and motile sperm, along with decreased percentages of dead and abnormal sperm. Despite these improvements, the percentages of live, normal, and motile sperm in the busulfan + propolis group remained significantly lower than those of the control group, though sperm count and the percentage of abnormal sperm were normalized (Table 3).

**TABLE 3:** Effects of propolis on the semen parameters in busulfan intoxicated rats.

Group Parameter	Control	DMSO	Busulfan	Propolis	Busulfan + propolis	P value
Sperm count (10^6^/mL)	44.000 ± 10.060^ab^	51.750 ± 9.716^a^	3.000 ± 0.577^c^	34.600 ± 4.130^ab^	25.600 ± 2.441^b^	0.001
Dead sperm (%)	71.804 ± 6.940^b^	64.000 ± 2.160^b^	139.690 ± 6.545^a^	24.625 ± 4.284^c^	35.000 ± 2.887^c^	0.000
Alive sperm (%)	188.750 ± 12.286^a^	162.500 ± 10.120^b^	47.308 ± 3.784^d^	72.000 ± 2.000^c^	77.000 ± 6.633^c^	0.000
Normal sperm (%)	264.400 ± 6.470^a^	236.000 ± 9.513^b^	40.833 ± 1.537^d^	68.571 ± 11.686^c^	70.000 ± 3.652^c^	0.000
Abnormal sperm (%)	29.500 ± 2.156^b^	36.750 ± 2.005^b^	48.800 ± 6.012^a^	12.500 ± 1.890^c^	30.000 ± 4.472^b^	0.000
Motility (%)	75.000 ± 2.739^a^	71.667 ± 4.410^a^	13.000 ± 2.000^d^	58.333 ± 5.578^b^	40.833 ± 3.270^c^	0.000

Data are expressed as the mean ± SE (n = 8).

^abcd^ different letters indicate significant difference at P<0.05.

### 3.4 Propolis improved the testicular redox balance in busulfan-exposed rats

No significant differences were observed in the studied redox parameters between the control and DMSO groups, except for a significant decrease in testicular NO levels in the DMSO group compared to the control. Busulfan exposure caused a notable disturbance in the oxidant/antioxidant balance, as evidenced by a significant increase in testicular MDA levels in concomitant with significant decreases in testicular NO and GSH levels, CAT and SOD activities, and serum TAC compared to the control group. A similar pattern was observed between the busulfan and DMSO groups, except for testicular NO levels, which remained unchanged between these groups. Propolis administration resulted in a significant decrease in testicular NO levels, SOD and CAT activities, and serum TAC, combined with a significant increase in testicular GSH levels, with no significant change in testicular MDA levels compared to the control group. A similar pattern was observed between the propolis and DMSO groups, except for testicular NO levels, which showed no significant difference between these groups. No significant differences in redox parameters were detected between the propolis and busulfan groups, except for a significant increase in testicular GSH levels in the propolis group compared to the busulfan group. Supplementation with propolis in busulfan-intoxicated rats markedly improved redox status, as indicated by a significant decrease in testicular MDA levels and significant increases in testicular NO and GSH levels, CAT and SOD activities, and serum TAC compared to the busulfan group. All studied redox parameters returned to control levels, except for testicular CAT activity, which remained significantly lower than control levels (Table 4).

**TABLE 4:** Effects of propolis on the testicular redox parameters and plasma total antioxidant capacity in busulfan intoxicated rats.

Group Parameter	Control	DMSO	Busulfan	Propolis	Busulfan + propolis	P value
Testicular MDA level (nMol/mg protein)	0.130 ± 0.015^b^	0.111 ± 0.006^b^	0.220 ± 0.024^a^	0.176 ± 0.022^ab^	0.143 ± 0.013^b^	0.014
Testicular NO level (µmol/mg protein)	9.268 ± 1.306^a^	4.569 ± 0.608^b^	3.453 ± 0.439^b^	4.344 ± 0.673^b^	8.482 ± 1.300^a^	0.001
Testicular CAT activity (U/mg protein)	1.040 ± 0.040^a^	0.926 ± 0.236^a^	0.125 ± 0.008^c^	0.127 ± 0.005^c^	0.554 ± 0.065^b^	0.000
Testicular SOD activity (mg/mg protein)	0.152 ± 0.004^a^	0.154 ± 0.003^a^	0.0513 ± 0.006^b^	0.064 ± 0.004^b^	0.128 ± 0.022^a^	0.000
Testicular GSH level (mg/mg protein)	0.620 ± 0.033^b^	0.604 ± 0.039^b^	0.364 ± 0.040^c^	0.750 ± 0.042^a^	0.630 ± 0.034^b^	0.000
Plasma TAC (mM/L)	0.871 ± 0.106^a^	0.668 ± 0.140^ab^	0.229 ± 0.095^c^	0.357 ± 0.144^bc^	0.715 ± 0.033^a^	0.003

MDA, malondialdehyde; NO, nitric oxide; CAT, catalase; SOD, superoxide dismutase; GSH, reduced glutathione; TAC, total antioxidant capacity.

Data are expressed as the mean ± SE (n = 8).

^abcd^ different letters indicate significant difference at P<0.05.

### 3.5 Histological features of testis were enhanced by propolis supplementation in busulfan-exposed rats

**FIGURE 1:**
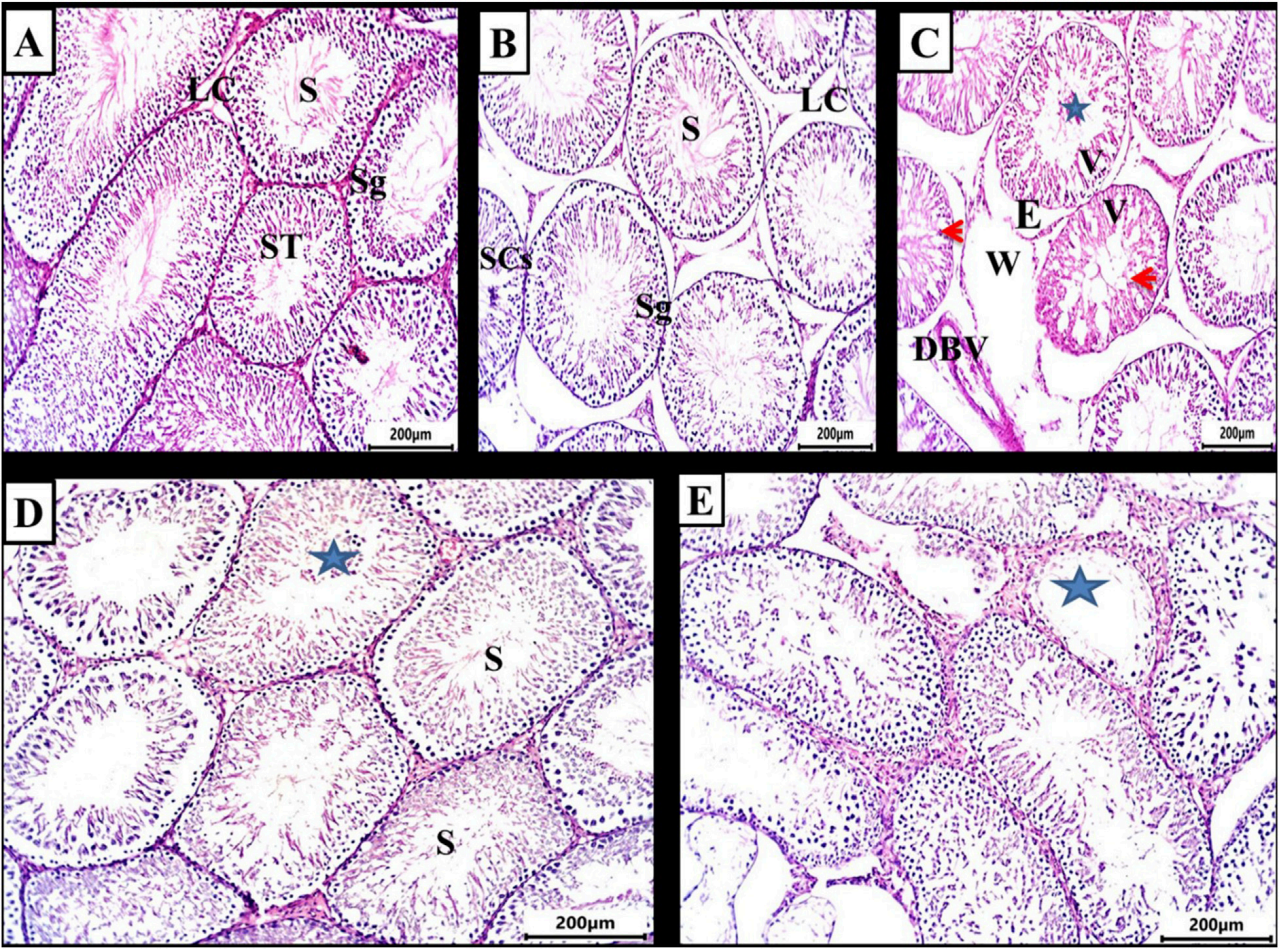
Photomicrograph of testis from rat (6 weeks). **(A,B)** control and DMSO groups showing a normal shape of seminiferous tubules (ST) and in between there were numerous interstitial cells of Leydig (LC). The seminiferous tubules were lined by stratified germinal epithelium which represents the spermatogenic cells (Sg) in different stages of development up to mature sperm. Large primary spermatocyte (PS), round spermatid, elongated spermatid, and late-stage sperm (S) attached to the apices of Sertoli cells (SCs). **(C)** Busulfan group showing the shrunken tubules surrounded by edema **(E)**, vacuolation of cells (V), widening of inter-tubular spaces were observed (W), absences of regular spermatogenesis, many tubules contain aggregated cells in their core (star) with degenerated sperm and dilated blood vessel (DBV). **(D)** Propolis extract-treated group showing seminiferous tubules lined with uniformly arranged spermatogenic cells, many tubules contain aggregated cells in their core (star) and sperm bundles in the lumen of seminiferous tubules (s). **(E)** Busulfan + Propolis group showed mild degenerative changes of some seminiferous tubules (stars). Most of the tubules were typically normal with different degrees of spermatogenesis. (H&E stain scale bar 200 µm).

Figures 1A,B illustrate the testes in both the control and DMSO groups, displaying the normal structure of seminiferous tubules. Numerous interstitial Leydig cells were present between the tubules, which were lined with stratified germinal epithelium. The spermatogenic cells were observed at various stages of development, including large primary spermatocytes, round spermatids, elongated spermatids, and late-stage sperm attached to the apices of Sertoli cells. In contrast, testicular sections from the busulfan group showed significant histopathological alterations, such as shrunken tubules surrounded by edema, cellular vacuolation, widened inter-tubular spaces, and disrupted spermatogenesis. Many tubules exhibited aggregated cells in their core, degenerated sperm, and dilated blood vessels (Figure 1C). In the propolis extract group, seminiferous tubules were lined with uniformly arranged spermatogenic cells. Several tubules contained aggregated cells in their core, and sperm bundles were present in the lumens of seminiferous tubules (Figure 1D). In the Busulfan + Propolis group, marked improvement was observed. Most seminiferous tubules appeared normal with active spermatogenesis at varying stages (Figure 1E) indicating amelioration of many busulfan-induced alterations.

### 3.6 Propolis alleviated the intense PCNA and caspase-3 immunoexpression observed in the testes of busulfan-challenged rats

**FIGURE 2:**
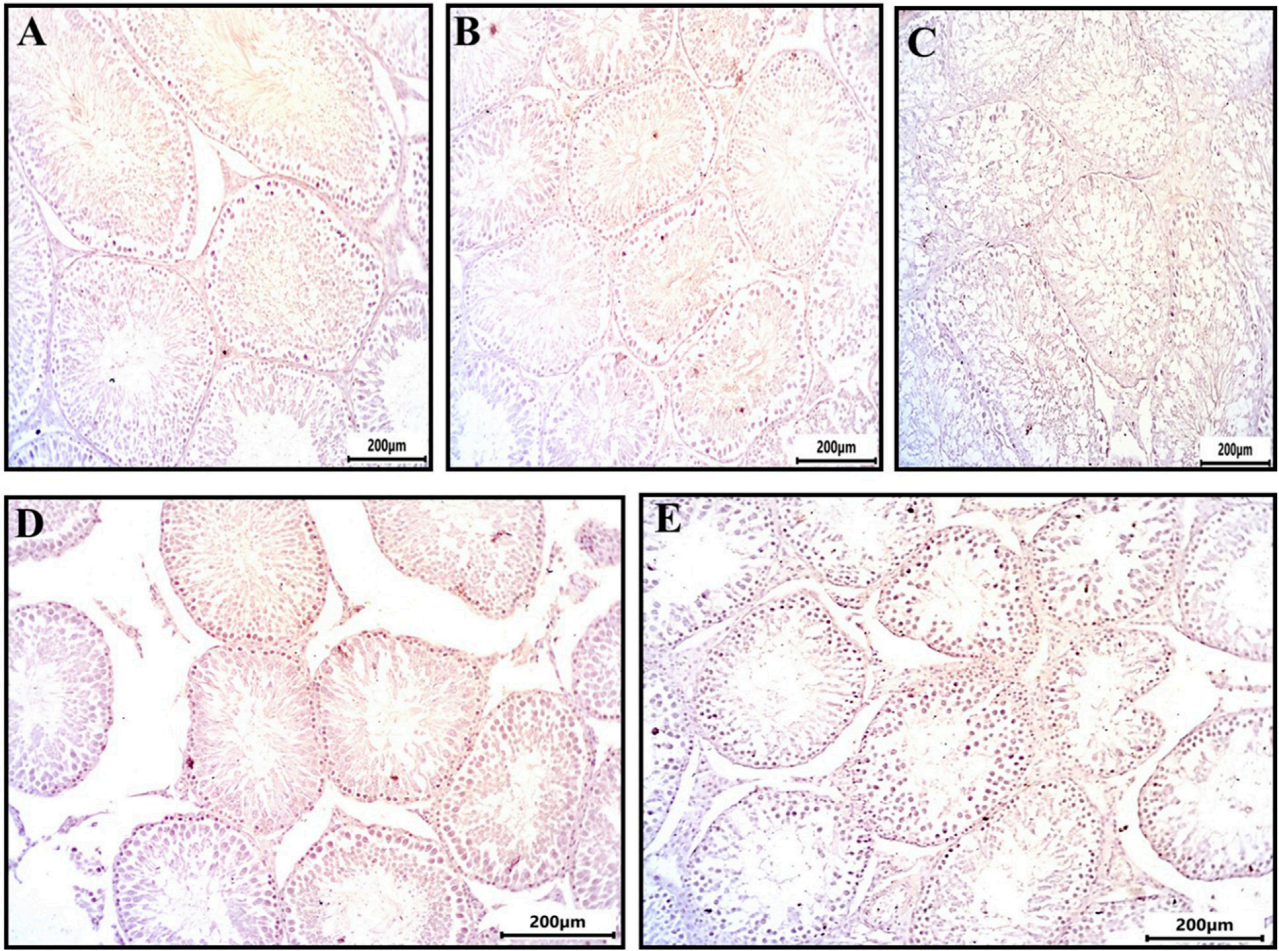
Sections from rat testis immune stained for PCNA (a marker of nuclear proliferation) showing all experimental groups: **(A)** control group showed no immunoreactivity for PCNA. **(B)** The DMSO group showed no immunoreactivity for PCNA. **(C)** Busulfan group showed strong immunoreactivity for PCNA indicated by brownish coloration in spermatogonia and spermatocytes. **(D)** Propolis group showed no immunoreactivity for PCNA. **(E)** Busulfan + Propolis group showed moderate immunoreactivity for PCNA. Photographed with Camera (N) X-100 (IHC, scale bar 200 µm).

Immunohistochemical analysis of PCNA expression in testicular sections revealed that spermatogonia, primary spermatocytes, secondary spermatocytes, and spermatids in the control, DMSO, and Propolis groups exhibited minimal to no PCNA staining (Figures 2A,B,D). In contrast, the Busulfan group demonstrated strong immunoreactivity for PCNA (Figure 2C), while the Busulfan + Propolis group showed moderate PCNA immunoreactivity (Figure 2E).

**FIGURE 3:**
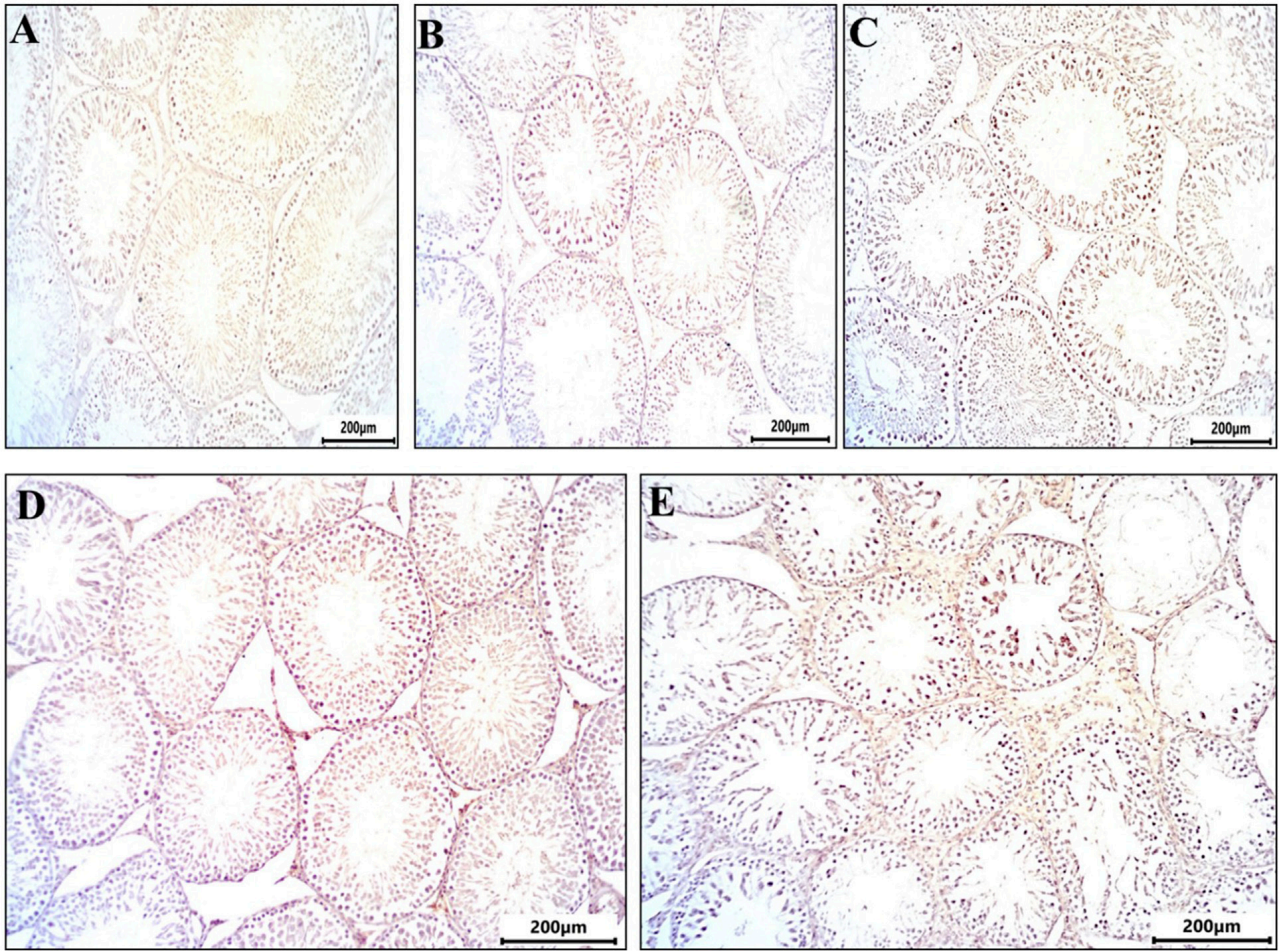
Sections from rat testis immune stained for Caspase 3 (marker programmed cell death (apoptosis) showing all experimental groups: **(A)** control group showed no immunoreactivity for caspases. **(B)** The DMSO group showed no immunoreactivity for caspases similar to control. **(C)** Busulfan group showed strong immunoreactivity for caspases indicated by brownish coloration in spermatogonia and spermatocytes. **(D)** Propolis showed mild immunoreactivity for caspases indicated by brownish coloration in some spermatocytes compared to control. **(E)** Busulfan + Propolis group showed moderate immunoreactivity for caspases indicated by brownish coloration in some spermatogonia, and spermatocytes. Photographed with Camera (N) X-100 (IHC, scale bar 200 µm).

**FIGURE 4:**
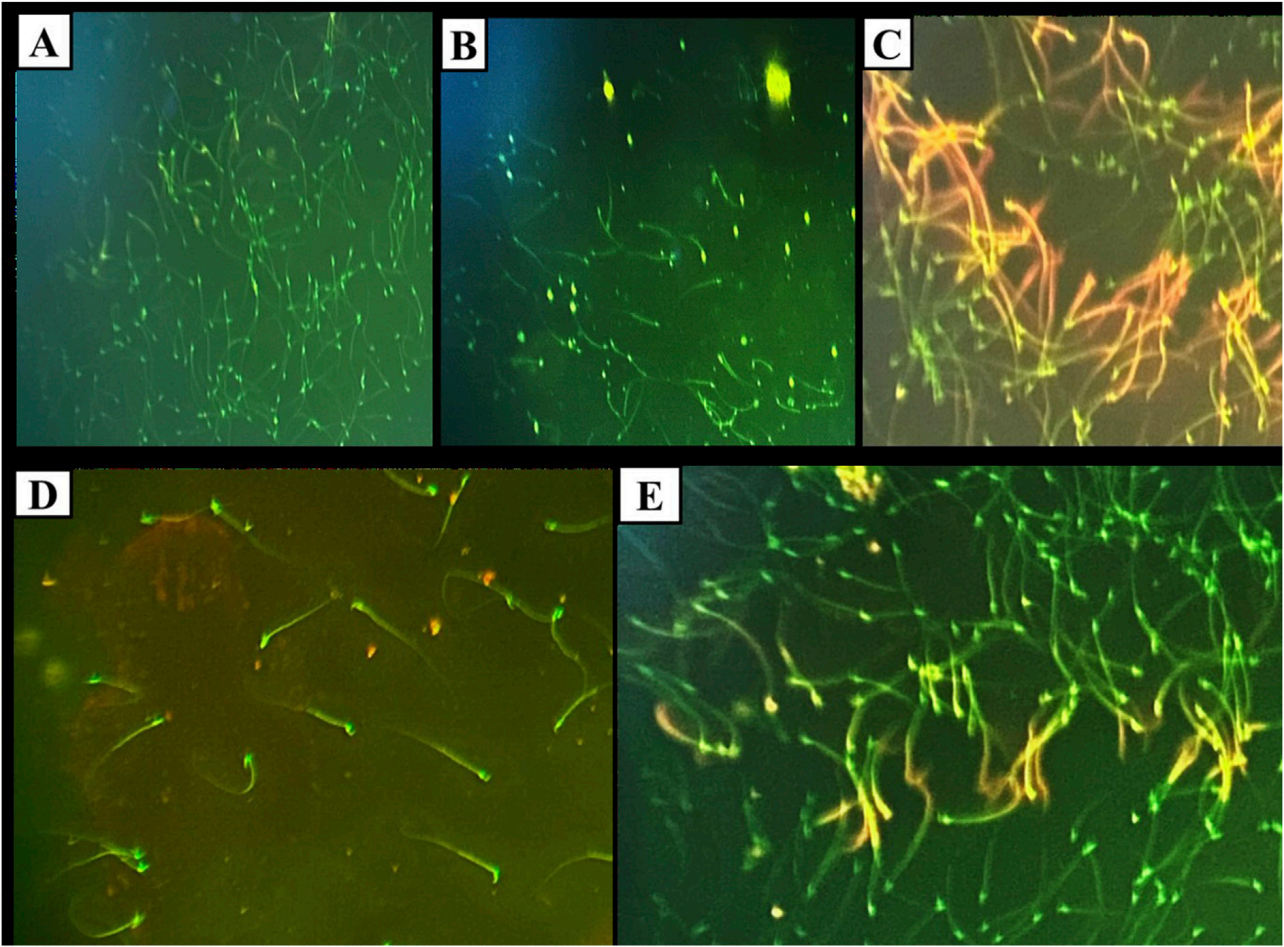
DNA Integrity assay using Acridine orange stain under the fluorescent microscope showing sperms in all experimental groups: **(A)** The control group shows green sperms ranked as spermatozoa with intact DNA. **(B)** The DMSO group shows green sperms ranked as spermatozoa with intact DNA similar to the control. **(C)** Busulfan group, green sperms ranked as spermatozoa with intact DNA, and orange and yellow sperms ranked as DNA-fragmented spermatozoa. Note the count of spermatozoa with green heads is small compared to spermatozoa with yellow or orange redheads. **(D)** Propolis group, green sperms ranked as spermatozoa with intact DNA. **(E)** Busulfan + Propolis group, green sperms ranked as spermatozoa with intact DNA, and orange and yellow sperms ranked as DNA-fragmented spermatozoa. Note the count of spermatozoa with green heads is extensive compared to spermatozoa with yellow or orange redheads. (Magnification power ×40).

Figure 4 depicts testicular sections immunostained for caspase-3, a marker of programmed cell death. The control and DMSO groups displayed no immunoreactivity for caspase-3 (Figures 3A,B). In the Busulfan group, strong immunoreactivity for caspase-3 was observed, characterized by brownish staining in spermatogonia and spermatocytes (Figure 3C). The Propolis group showed mild caspase-3 immunoreactivity, with brownish staining observed in some spermatocytes compared to the control (Figure 3D). The Busulfan + Propolis group demonstrated moderate caspase-3 immunoreactivity, with brownish staining visible in some spermatogonia and spermatocytes (Figure 3E).

### 3.7 The busulfan-induced genotoxicity and morphological alterations were counteracted by propolis intervention in the sperms of rats

**FIGURE 5:**
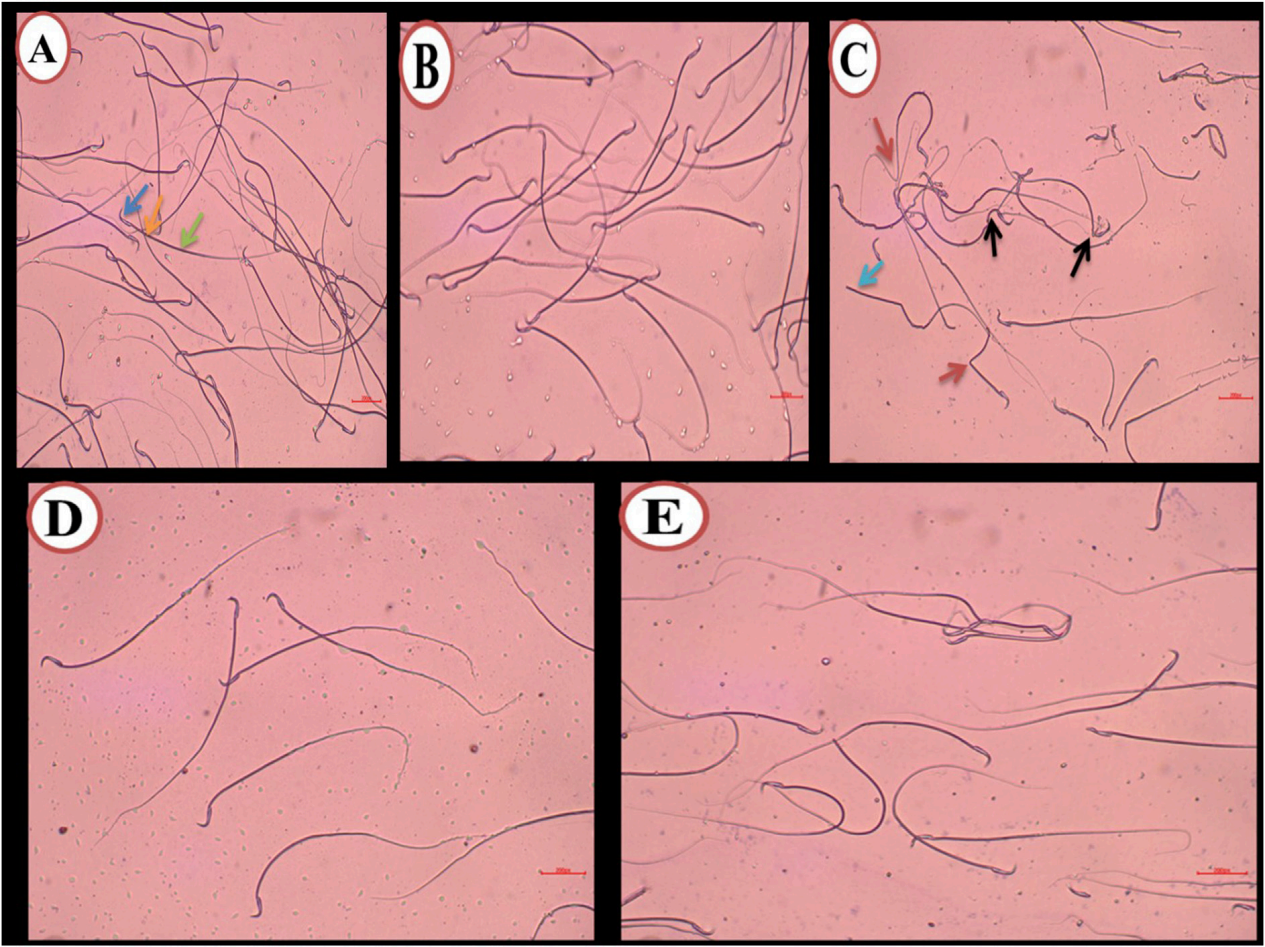
Methyl violet and sodium carbonate-stained semen film purple **(A)** of control rats showing normal epididymal sperms structure, (blue arrow) hooked head, (orange arrow) midpiece, and (green arrow) single long tail. **(B)** The DMSO group during the experiment showed normal sperm morphology the same as the control group. **(C)** The Busulfan group shows a lot of sperm abnormalities, (black arrow) Bent neck, (blue arrow) Headless, and (red arrow) curvature tail. **(D)** The Propolis group shows normal sperm as the control group. **(E)** The Busulfan + Propolis group shows few abnormalities compared to the busulfan group. (Scale bar 200 µm).

**FIGURE 6:**
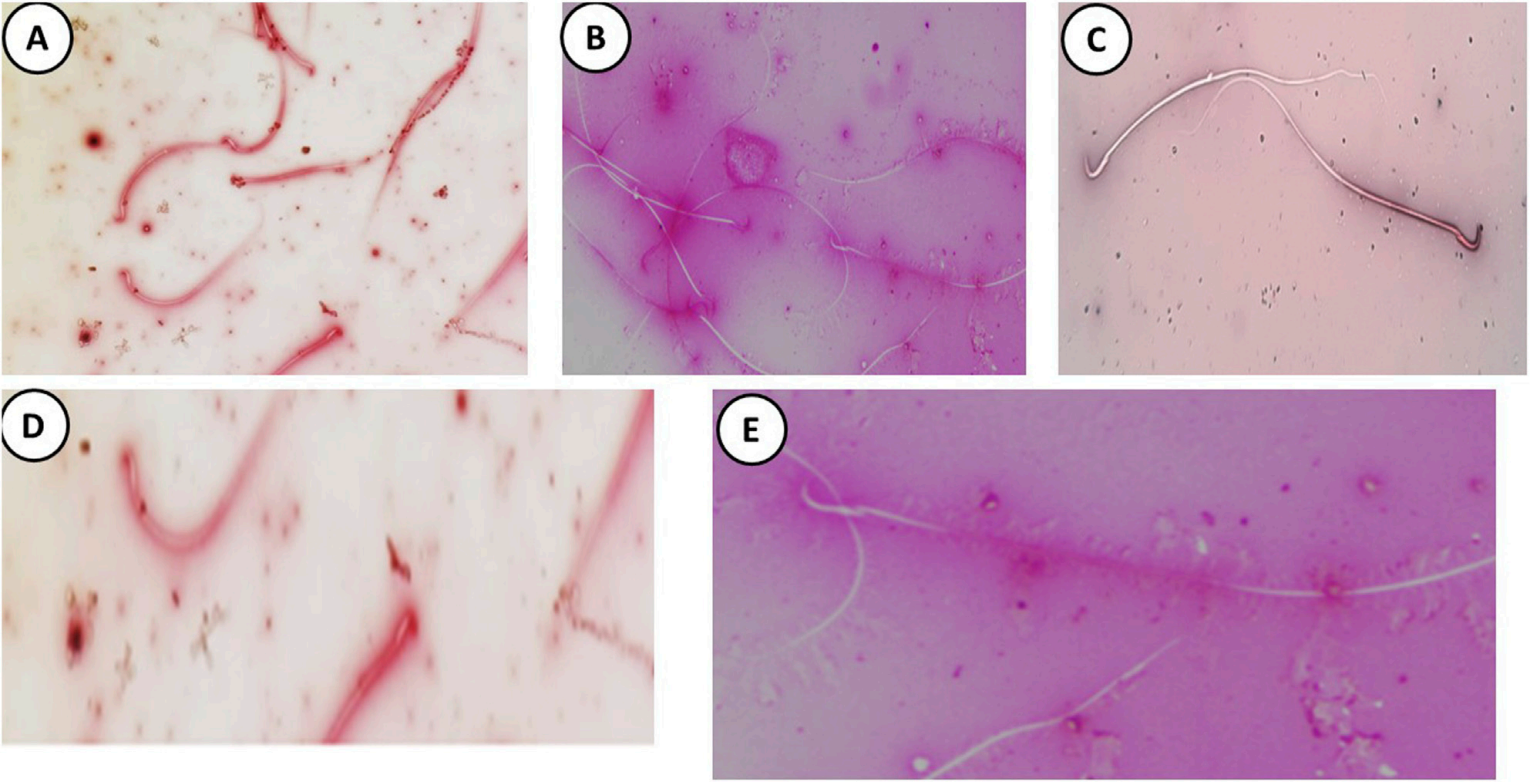
Eosin and Nigrosine-stained semen **(A)** film of control rats showing normal alive epididymal sperms represented as unstained head under a light microscope. **(B)** The DMSO group during the experiment showed unstained sperm the same as the control group. **(C)** The Busulfan group shows an alive unstained head and dead epididymal sperms represented with pink heads (green arrow). **(D)** The Propolis group shows normal sperm as the control group. **(E)** The Busulfan + Propolis group showed an increase in the number of alive epididymal sperms with unstained heads compared to the busulfan group. (Scale bar 200 µm).

The DNA integrity assay using Acridine Orange stain revealed that the control, DMSO, and Propolis groups predominantly exhibited green spermatozoa, indicating intact DNA (Figures 4A,B,D). In the Busulfan group, green spermatozoa with intact DNA were still observed; however, a notable increase in spermatozoa with fragmented DNA, represented by orange and yellow fluorescence, was evident. The proportion of spermatozoa with green heads was significantly lower compared to those with yellow or orange-red heads (Figure 4C). In the Busulfan + Propolis group, both green spermatozoa with intact DNA and fragmented DNA spermatozoa (orange and yellow) were present, with an increased proportion of spermatozoa with green heads compared to the Busulfan group (Figure 4E). The control, DMSO, and Propolis groups showed normal epididymal sperm structures, including a well-defined midpiece and single long tail (Figures 5A,B,D). In contrast, the Busulfan group exhibited significant sperm abnormalities, such as bent necks, headless sperm, and curved tails (Figure 5C). The Busulfan + Propolis group demonstrated a marked reduction in sperm abnormalities compared to the Busulfan group (Figure 5E). In the control, DMSO, and Propolis groups, live epididymal spermatozoa were characterized by unstained heads (Figures 6A,B,D). The Busulfan group displayed a mixture of live spermatozoa with unstained heads and dead spermatozoa with pink-stained heads (Figure 6C). The Busulfan + Propolis group showed an increased number of live spermatozoa with unstained heads compared to the Busulfan group (Figure 6E).

## 4 Discussion

Busulfan intoxication causes a significant disruption in pituitary-gonadal hormone levels, consistent with the findings of Rostami et al. (2022). This disruption is attributed to decreased secretion of gonadotropin-releasing hormone, downregulation of gonadotropic receptors, and the loss of functional Leydig cells (Whirledge et al., 2015; Kim et al., 2023; Khamis et al., 2023). Busulfan-induced redox imbalance likely compromises the functional integrity of the hypothalamic-pituitary-gonadal axis (Spiers et al., 2015). The reduction in FSH impairs the release of androgen-binding protein, destabilizing intratubular testosterone concentrations (Mohlala et al., 2023). This further affects the synthetic capacity of Leydig cells by disrupting the function of proteins such as the steroidogenic acute regulatory protein, which mediates cholesterol transport into mitochondria (Leisegang and Henkel, 2018). The loss of testosterone’s negative feedback on LH secretion results in elevated LH levels (Abarikwu et al., 2022).

The restoration of hormonal balance in the pituitary-gonadal axis aligns with findings in cadmium-intoxicated rats (Amr et al., 2024). The elevated testosterone levels were linked to the upregulation of LH receptor expression and enhanced activity of steroidogenic enzymes (Nna et al., 2020). The elevation in serum FSH levels may be linked to propolis’s ability to reduce oxidative stress and enhance antioxidant capacity in the hypothalamus and pituitary gland (Emil et al., 2024). In addition, propolis may provide energy to the hypothalamus, facilitating the production of GnRH, which stimulates the anterior pituitary to release FSH from gonadotroph cells (Handayani and Gofur, 2019). Our GC-MS analysis revealed that propolis contains amino acids like glycine and flavonoids such as apigenin, which have been shown to enhance the expression of genes associated with testosterone synthesis (Ommati et al., 2022; Wu et al., 2024).

The adverse effects of busulfan on semen quality align with the findings of Kim and his colleagues (2023). These effects are attributed to the inhibition of the meiotic process in spermatogenic cells and stem cell self-renewal, induction of apoptosis, excessive free radical production, cytological damage to seminiferous tubules, disruption of the blood-testis barrier, and degeneration of sperm cytoskeleton (Li et al., 2023; Wang et al., 2024; Yue et al., 2024). The low sperm viability and motility may be owed to the low level of ATP (Abd-Elrazek et al., 2020). The deficiency in testosterone as a primary androgen responsible for spermatogenesis is a key factor in disruption in semen profile (Smith and Walker, 2014). The plasma membrane of the sperm is particularly vulnerable to oxidative insult due to its high content of polyunsaturated fatty acids and limited antioxidant enzyme activity (Leisegang and Henkel, 2018; Mohlala et al., 2023). The busulfan-evoked lipid peroxidation leads to damage to the axoneme, an essential component for sperm motility, in addition to increased morphological abnormalities and reduced sperm viability, ultimately inhibiting spermatogenesis and resulting in a decreased sperm count (Hussain et al., 2023; AbdElrazek et al., 2024). It also evoked DNA fragmentation in germ cells in corresponding to a previous report (Bahmyari et al., 2024).

The improvement in sperm measurements corroborates a previous peer-reviewed article (Seven et al., 2020) due to rebalance of pituitary-gonadal axis, improvement in steroidogenesis, increase in energy resources availability for germ cell development and reduction in intra-testicular oxidative insult and apoptosis of germ cells (Nna et al., 2020). Propolis had the ability to enhance sperm motility by augmenting the overall mitochondrial respiratory efficiency of spermatozoa *in vitro* (Cedikova et al., 2014). The preservation of genomic material in spermatozoa is similar to the observation of Abd-Elrazek et al. (2020) as confirmed by the ability of propolis to protect against DNA strand break under free radical attack (Guzelmeric et al., 2023).

Our study’s findings demonstrate the significant protective effects of propolis against busulfan-induced reproductive dysfunction, evidenced by the restoration of hormonal profiles, improvement in sperm quality, mitigation of oxidative stress, and alleviation of histopathological changes. However, a closer examination of the propolis-alone group revealed certain statistically significant differences in reproductive hormones (decreased LH, testosterone, and E2) and sperm parameters (decreased percentages of dead, live, normal, abnormal, and motile sperm) compared to the control group. At first glance, these observations might appear contradictory to propolis’s overall therapeutic role. We contend that these findings reflect the complex, context-dependent, and adaptogenic properties of propolis, rather than inherent reproductive toxicity in healthy animals.

Propolis, a rich mixture of bioactive compounds including flavonoids, phenolic acids, and terpenes, is known to exert diverse pharmacological actions (Hossain et al., 2022). Its constituents can interact with various physiological pathways, including those involved in endocrine regulation. For instance, some flavonoids present in propolis, such as chrysin, have been investigated for their potential to influence aromatase activity, an enzyme crucial for estrogen synthesis from androgens (Balam et al., 2020). Such interactions can subtly modulate the intricate feedback loops governing the hypothalamic-pituitary-gonadal axis, leading to a new physiological equilibrium rather than a pathological state. These shifts in hormone levels in a non-stressed system may represent a re-calibration by propolis’s active components to optimize endocrine balance, as opposed to inducing dysfunction.

Similarly, the alterations in sperm parameters observed in the propolis-alone group, where there was a reduction in both dead and abnormal sperm alongside a slight decrease in live, normal, and motile sperm, can be interpreted within the context of physiological adaptation and quality control. Propolis possesses potent antioxidant and anti-apoptotic properties (Mohamed et al., 2022). In a healthy testis, there is a natural, ongoing process of germ cell turnover and quality assurance, which involves a degree of physiological apoptosis to eliminate defective cells (Asadi et al., 2021). It is plausible that propolis, by enhancing the cellular antioxidant defense and mitigating basal cellular stress, subtly influences this intrinsic quality control mechanism. This could lead to a more “pruned” population of spermatozoa, where fewer defective cells are present, even if it results in a minor adjustment in other parameters in an otherwise healthy system. This suggests a potential for optimizing sperm quality rather than causing outright damage.

The critical distinction lies in the adaptogenic nature of propolis, a characteristic shared by many traditional medicinal compounds. Adaptogens are substances that help the body adapt to various stressors and normalize physiological functions. Their effects are often highly dependent on the organism’s prevailing homeostatic state (Panossian and Wikman, 2010; Panossian et al., 2021). In a diseased or severely stressed system, such as the busulfan-induced reproductive dysfunction model, propolis’s robust redox-stabilizing, anti-inflammatory, and cytoprotective properties become paramount. Here, propolis acts as a potent restorative agent, actively counteracting the overwhelming damage, oxidative stress, and inflammation caused by busulfan, effectively bringing perturbed parameters back towards normal physiological levels (Ayad et al., 2025). Conversely, in a healthy, non-stressed system, propolis’s influence shifts from “restoration” to subtle “modulation” or “optimization” of existing physiological pathways. These modulations, while statistically distinct from the control baseline, do not necessarily equate to toxicity but rather represent an adaptive response to the presence of its bioactive compounds, guiding the system towards an optimized homeostatic set point (Sforcin and Bankova, 2011; El-Guendouz et al., 2019).

Therefore, the apparent contradiction resolves when considering propolis’s capacity to function differently based on the physiological context. Its effects are not merely unidirectional but are instead dynamically adjusted to the needs of the organism, promoting either repair in compromised states or fine-tuning in healthy ones.

A wide range of experimental models for testicular dysfunction highlights the redox-disrupting effects of busulfan (Qian et al., 2020; Abarikwu et al., 2022). This disruption is attributed to busulfan’s ability to reduce electron flow through respiratory chain complex I, accelerate the production of intracellular reactive oxidants, downregulate enzymatic antioxidants, and suppress redox-related transcription factors (Rigobello et al., 1999; Li et al., 2018; Afzali et al., 2022). The depletion of testicular antioxidants arises from the heightened demand to neutralize reactive free radicals, which diminishes the cell’s capacity to counteract oxidative damage effectively, leaving these cells vulnerable to cytological injury (Kwon et al., 2019).

Redox rebalance in the testicular tissue of busulfan-intoxicated rats following propolis administration is corresponding to the findings of Demir et al. (2023). According to our GC-MS analysis, this response can be explained by presence of flavonoids and phenolic compounds which possess potent antioxidant ability by donating electrons to reactive oxidants, chelating metal ions, and stimulating antioxidant and detoxifying enzymes (Ozkan et al., 2017; Demir et al., 2018). The amino acids found in propolis confer antioxidant shield. For instance, glycine is incorporated as an essential building block in biosynthesis of GSH, and L-cystine acts as an important player in Nrf2-involved redox signaling (Howard et al., 2010; Dai and Chen, 2023). Flavonoids such chrysin and chalcones possess free radical scavenging properties (Iqbal et al., 2014; Mani and Natesan, 2018). The improvement in the activity of redox stabilizers advantages the testicular functionality. NO plays a crucial role in enhancing testicular blood flow, promoting testosterone synthesis, and supporting spermatogenesis (Middendorff et al., 1997; El-Migdadi et al., 2005; Christin-Maitre and Young, 2022). GSH improves the morphology of seminiferous tubules and facilitates spermatogenesis (Abdullah et al., 2021; Chen et al., 2023). SOD and CAT safeguard spermatozoa from oxygen free radicals and protect their lipid membranes from peroxidation (Asadi et al., 2017).

Histopathological deteriorations associated with busulfan are in consistent with the findings of Abd El-Hay et al. (2023), confirming its cytotoxic nature and its ability to arrest spermatogenesis. The absence of favorable growth factors, a supportive hormonal milieu, intact junctions, and appropriate signaling pathways after chemotherapy administration underlies the disruption of spermatogenesis (Anand et al., 2016). Busulfan exerts toxic effects on tissues through the irreversible alkylation of intracellular biomolecules (Nasimi et al., 2016). The reactive oxygen species generated by busulfan can damage critical biomolecules such as DNA and lipids. The widening of inter-tubular spaces often attributed to the shrinkage of seminiferous tubules (Rostami et al., 2022). The later could be due to progressive detachment of Sertoli cells, and loss of the structural integrity of the basal compartment, leading to a sort of tubular atrophy (Vasiliausha et al., 2016). The detachment of germ cells into the tubular lumen indicates a disruption in inter-Sertoli junctional complexes (Ibrahim et al., 2021). Busulfan also induces significant vacuolation within spermatogonial tubules, reflecting the inhibition of the meiotic process in spermatogonial cells and impairing cytoplasmic extrusion mechanisms (Malekinejad et al., 2011; Yue et al., 2024). These cellular vacuolations may represent debris and spaces left by the extensive death of germ cells (Fotouh et al., 2018). Inflammatory responses triggered by busulfan result in edema surrounding the seminiferous tubules, which subsequently interferes with testicular venous drainage and leads to inter-tubular capillary congestion (Mitchell, 2015).

The improvement in the histological features of the testes in busulfan-compromised rats co-administered with propolis aligns with findings in a rat model exposed to another chemotherapeutic agent, doxorubicin (Alsyaad, 2024). The cytoprotective properties of propolis against testicular dysfunction stem from its antioxidant properties, anti-apoptotic activity, and its ability to promote cell viability (Seven et al., 2021; Ashour, 2024; Duarte, 2024).

Our findings, along with previous studies (Sönmez et al., 2016; Abd El-Hay et al., 2023), demonstrate an increase in the immuno-expression of caspase-3 and PCNA in germ cell layer. Busulfan upregulates the adaptor protein, leading to significant alterations in mitochondrial membrane potential and the release of cytochrome C from the mitochondria into the cytoplasm, which activates apoptosis-related gene expression (Lindsten et al., 2000; Antonsson, 2001; Zhivotovsky and Kroemer, 2004). Busulfan triggers both intrinsic and extrinsic apoptotic pathway through tumor suppressor P53 and Fas receptor induction (Furukawa et al., 2007; Chen et al., 2018). Consistent with our outcomes, busulfan induces oxidative stress in the testis, which may contribute to oxidative damage to DNA histones and disrupt DNA repair enzyme expression, ultimately triggering apoptosis (Sifuentes-Franco et al., 2018). Exposure to busulfan causes DNA cross-linking, DNA–protein crosslinking, and single-strand breaks, which block DNA replication and transcription, inhibiting cell proliferation (Chen et al., 2018). The elevated testicular levels of the proliferative marker in busulfan-treated rats, compared to control rats, may represent a compensatory response to impaired spermatogenesis (Westernströer et al., 2014), similar to findings reported with another gonadotoxic chemotherapy, cisplatin (Elrashidy and Hasan, 2020). However, this response appears insufficient to repair the testicular damage induced by busulfan exposure.

The decreased immuno-expression of caspase-3 in the testes of busulfan-intoxicated rats following propolis intervention aligns with findings by Seven and his coauthors (2020). The bioactive compounds in propolis, such as flavonoids, have been shown to inhibit apoptosis by downregulating Bax and caspase-3 expression while upregulating Bcl-2 expression (Zhang et al., 2025). The reduced the expression of proliferation marker PCNA in the testis of busulfan + propolis group is in harmony with a previous peer-reviewed article (Sönmez et al., 2016). Our findings highlight the potential of propolis to restore the balance between proliferation and apoptosis within the testicular microenvironment, which plays a crucial role in maintaining normal spermatogenesis (Zakariah et al., 2022).

The comprehensive GC-MS analysis of the propolis extract used in this study revealed a rich and diverse phytochemical profile (Table 1), comprising fatty acid esters, aromatic compounds, amino acid derivatives, saccharides, and notably, a significant proportion of flavonoids and terpenes. This intricate chemical composition underpins the multifaceted biological activities observed, particularly the potent antioxidant and anti-apoptotic effects that mitigate busulfan-induced reproductive dysfunction (Ye et al., 2020; Ileriturk et al., 2021; Demir et al., 2024).

The primary drivers of the observed antioxidant and anti-apoptotic effects are predominantly attributed to the high concentrations of flavonoids and certain phenolic compounds identified in our propolis sample, along with contributions from terpene derivatives and specific amino acids (El-Guendouz et al., 2019; Belmehdi et al., 2023).

Specifically, our analysis identified several key flavonoids, including Pinostrobin chalcone (7.793%), Tectochrysin (8.162%), Pinocembrin (3.843%), Apigenin 7-methyl ether (1.409%), and Chrysin (2.014%). These flavonoids are renowned for their robust free radical scavenging capabilities, ability to chelate metal ions, and direct modulation of antioxidant enzyme activities (Lan et al., 2016; Ileriturk et al., 2021; Ali Abed Wahab et al., 2023; El Menyiy et al., 2023; Ijaz et al., 2024). Furthermore, flavonoids like chrysin have been extensively studied for their anti-apoptotic properties. They can intervene in apoptotic pathways by modulating the expression of pro-apoptotic proteins (e.g., caspase-3, as indicated by our immunohistochemistry results) and anti-apoptotic proteins (e.g., Bcl-2 family members), and by mitigating oxidative stress, thereby preserving cellular integrity and viability (Soliman et al., 2022; Şimşek et al., 2023). Tectochrysin and pinocembrin also contribute significantly, with studies demonstrating their ability to reduce oxidative damage and protect cells from apoptosis induced by various toxins (Ijaz et al., 2024; Darwish et al., 2025).

Beyond flavonoids, other identified compounds also contribute to the observed effects. The presence of Beta-monostearin (9.865%) and 2-Hexadecanoyl glycerol (22.976%), which are glycerol derivatives, can play a role in membrane stability and cellular signaling, indirectly supporting cytoprotection (Showalter et al., 2020). Terpene derivatives such as α-Curcumene (3.74%) and α-Eudesmol (2.682%) also possess notable antioxidant and anti-inflammatory activities, which would synergistically contribute to the overall protective effects against busulfan-induced damage by reducing oxidative burden and inflammatory responses (Liju et al., 2011; Acharya et al., 2021). Additionally, the identification of amino acids like L-Cystine (0.314%), Asparagine (0.448%), and Glycyl-D-asparagine (1.024%) is noteworthy. L-Cystine, in particular, is a precursor to GSH, a master endogenous antioxidant, and its presence suggests a potential contribution to the restoration of reduced glutathione levels observed in our study, thereby directly bolstering the testicular defense system (Justin Margret and Jain, 2024). The significant presence of 3-O-Methyl-D-glucose (12.545%), a monosaccharide derivative, directly contributes to protecting cells from stress-induced damage and supporting their viability (Zou et al., 2024).

In conclusion, propolis reversed the hypergonadotropic-hypogonadism induced in busulfan-challenged rats by improving the deteriorated testicular architecture, enhancing sperm count and quality, restoring the testicular antioxidant defense system, and reestablishing the dynamic equilibrium of the reproductive hormonal axis. This study highlights propolis as a promising candidate for mitigating the cytotoxic effects of busulfan on testicular homeostasis. However, the differences in testicular architecture between rodents and primates, including humans, should be carefully considered before translating these findings to clinical applications. While this study comprehensively demonstrates the ameliorative effects of aqueous propolis on busulfan-induced reproductive dysfunction at the physiological, biochemical, and histopathological levels, a more in-depth exploration of the underlying molecular mechanisms, particularly through techniques such as Western blotting for protein expression (e.g., PCNA, Caspase-3, StAR, AR) and quantitative real-time PCR for gene expression, was beyond the scope of the current research due to resource and infrastructure limitations. Future studies are warranted to fully elucidate these molecular pathways.

## Data Availability

The original contributions presented in the study are included in the article/supplementary material, further inquiries can be directed to the corresponding author.
